# Migraineurs with exercise-triggered attacks have a distinct migraine

**DOI:** 10.1186/1129-2377-14-99

**Published:** 2013-12-21

**Authors:** Hille Koppen, Peter LJ van Veldhoven

**Affiliations:** 1 Department of neurology, HagaZiekenhuis Teaching hospital, Leyweg 275, 2445 CH, The Hague, The Netherlands; 2 Department of sports medicine, Medical Center Haaglanden, Leidschendam, The Netherlands

**Keywords:** Migraine, Trigger, Sport, Exercise

## Abstract

**Background:**

Sport as a migraine trigger has been reported, but extensive information on these triggered attacks and the patients experiencing these attacks is lacking. Goal of this study was to investigate the lifetime prevalence of exercise triggered migraine attacks in migraine patients and if patients with exercise triggered attacks experience specific prodromal or ictal migraine symptoms.

**Methods:**

103 consecutive migraine patients seen during their first visit at a Dutch headache clinic were administered an interview during their first visit to the outpatient headache clinic in which they were asked about their normal life migraine characteristics and if exercise had ever triggered a migraine attack within 48 hours after stopping exercise. Those reporting exercise triggered migraine attacks, were asked if these migraine attacks were typical or atypical compared to their normal life attacks and which kind of exercise in particular could provoke migraine attacks.

**Results:**

Among migraineurs lifetime prevalence of exercise-triggered migraine attacks was 38%, regardless of migraine type (with or without aura) or gender. Neck pain as the initial migraine symptom during normal life migraine attacks, was more frequent in those experiencing exercise-triggered migraine attacks. More than half of the patients reporting exercise-triggered migraine attacks abandoned the offending sport due to migraine. As our study population was drawn from a headache clinic, result can not be generalized to the general population.

**Conclusions:**

Life time prevalence of exercise-triggered migraine attacks was high. Those experiencing exercise-triggered migraine attacks, more frequently had neck pain as initial migraine symptom during normal life attacks.

## Background

Migraine attacks can be triggered by a variety of stimuli and exercise is reported to be one of them [1,2]. However, there are also studies reporting that sport activity (e.g. regular cycling) does not influence migraine frequency [3], or that sport even is effective in migraine prevention [4]. Most studies reporting on sport as a migraine trigger however lack extensive information on migraine diagnosis, migraine history and migraine symptoms. As a result it is not clear which migraineurs have the highest risk of developing exercise-triggered migraine (ETM) attacks, and if these attacks resemble “normal life” migraine attacks. Furthermore it has never been investigated if patients reporting sport as a migraine trigger actually quit this offending sport, which would be likely if there is an actual causal relation.

The objective of this study was to investigate the lifetime prevalence of ETM attacks in migraineurs, and if these migraineurs share typical characteristics. Moreover, received treatments and impact of ETM were evaluated.

## Methods

The study design of this study was retrospective as migraine patients (diagnosed according to the International Classification of Headache Disorders ICHD-II) [5] were questioned about their migraine history. A questionnaire was taken by an experienced headache neurologist (HK). Patients were asked about their normal life migraine characteristics and if exercise had ever triggered a migraine attack within 48 hours after stopping exercise, according to the ICHD-II definition of a migraine trigger [5]. Patients reporting to have ETM attacks, were asked if these migraine attacks were typical or atypical compared to their normal life attacks. Patients were asked which kind of exercise in particular could provoke migraine attacks. Furthermore it was asked if specific activities like sexual intercourse, Valsalva maneuver or air plane travel could also trigger migraine attacks. High migraine frequency was defined as higher than mean, which was 3 or more attacks each month. In total 103 consecutive migraine patients seen during their first visit at the HagaZiekenhuis outpatient Headache center, The Hague, The Netherlands, during 2012 were included. The outpatient Headache clinic of the HagaZiekenhuis teaching hospital serves both as a secondary and tertiary headache center. No patients refused participation. MR imaging of the brain was obtained in all participants, as a standard procedure of first visit to the headache center. No underlying structural abnormalities causing headaches or exercise-induced headaches were found. The study was conducted according to the Helsinki declaration revised in 2008.

### Statistics

Chi Square or Fisher’s exact test where appropriate were used. Continuous variables were analyzed using Student t-test. P < 0.05 was considered significant. Multiple comparisons have been made and this was corrected for. For normal life migraine symptoms a Bonferroni correction was used; p < 0.007 was considered significant. For occupancies; p < 0.017 was considered significant. SPSS Statistics, IBM SPSS Statistics for Windows was used, Version 19.0. Armonk, NY: IBM Corp.

## Results

Characteristics of migraine patients are shown in Table 1. All patients were of Caucasian race, most were females (86%), and according to ICHD-II criteria [5] 45% had migraine with aura. Mean age was 39.5 years and mean attack frequency was 2.7 per month.

**Table 1:** Characteristics

**Patient characteristics**	
Number of participants	103
Migraine with aura	46 (45%)
Female gender	89 (86%)
Mean age	39.5 (SD 12.8, range 18–78)
Attack frequency/ month, mean	2.7 (SD 1.8, range 1–10)
Migraine years, mean	19.8 (SD 13.3, range 1–60)
Migraine onset age, mean	19.5 (SD 9.9, range 4–57 )
MR imaging Brain normal	103 (100%)
Lifetime prevalence of ETM	38%

*Abbreviations*: *ETM* Exercise triggered migraine.

The lifetime prevalence of ETM attacks was 39/103 (38%), which did not differ between migraineurs with aura (35%) and migraineurs without aura (40%). Also no difference in ETM prevalence was found between males (50%) and females (36%). In 22/39 (56%) of participants with ETM the attacks started during exercise. In case the attack started after exercise, this was on average 160 minutes after the exercise was stopped (range 5–720 minutes). A total of 27/39 (69%) participants with ETM reported that these attacks were similar to their normal life migraine attacks. The remaining patients reported their ETM attacks to be more severe. More than half of ETM sufferers (21/39, 54%) reported to have quit the specific offending exercise because of ETM attacks. Only 7/39 (18%) of them reported to have ever used prescribed medication in order to prevent ETM. Sports and exercise that were mentioned to be associated with ETM attacks were running/cardio-fitness by 27/39, (69%) and racket sports (8/39, 21%). Head trauma accompanying sport as a trigger was mentioned by one patient, who could trigger migraine attacks by boxing.

### Normal life attacks

As shown in Table 2, of those migraineurs reporting ETM attacks, 13/39 (33%) reported neck pain as initial migraine symptom at the onset of normal life attacks, which was higher than in migraineurs without ETM attacks (9/64, 14%), p = 0.02. This association was strongest in males as neck pain as an initial symptom was never mentioned by non-ETM males. In the non-ETM group headache was most frequently reported as initial migraine symptom 20/64 (31%), vs 3/39 (8%) in the ETM group, p = 0.005. This was found to be even more apparent in females (30% vs 3%, p = 0.003).

**Table 2:** Migraine symptoms and history stratified for presence of ETM

	**ETM attacks (n = 39)**	**No ETM attacks (n = 64)**	**P-Value**
Age, mean	40.4	38.9	0.6
Migraine onset age, mean	18.4	20.1	0.4
Migraine frequency (per month), mean	2.9	2.5	0.2
Migraine history (years), mean	21.9	18.6	0.2
High attack frequency	20 (51%)	21 (33%)	0.06
*Migraine symptoms normal life attacks*			
Unilateral	34 (88%)	52 (81%)	0.4
Pulsatile	30 (77%)	45 (70%)	0.5
Nausea	36 (92%)	57 (89%)	0.6
Vomiting	21 (54%)	33 (52%)	0.8
Photophobia	33 (85%)	55 (86%)	0.9
Phonophobia	33 (85%)	55 (86%)	0.9
Aggravation by physical activity	39 (100%)	55 (86%)	0.013^#^
*Initial symptoms normal life attacks*			
Neck pain	13 (33%)	9 (14%)	0.02
Headache	3 (8%)	20 (31%)	0.005
Yawning	6 (15%)	8 (13%)	0.7
Aura	3 (8%)	11 (18%)	0.2
*Provocation of migraine by various occupancies*			
Airplane travel*	11 (28%)	6 (10%)	0.02&
Valsalva maneuver	4 (10%)	3 (5%)	0.3
Sexual intercourse	5 (13%)	4 (6%)	0.3
*Characteristics of ETM attacks*			
ETM starts during exercise	22 (56%)	N.a.	
ETM resembles normal life attack	27 (69%)	N.a.	
ETM causes quit of offending exercise	21 (54%)	N.a.	
Prescribed prophylaxis for ETM	7 (18%)	N.a.	

*Abbreviations*: *ETM* Exercise triggered migraine. *N.a.* not applicable.

Airplane travel provocation only in those who fly (n = 98)

^#^Bonferroni correction for multiple comparison, *p* < 0.007 considered significant.

&Bonferroni correction for multiple comparison, *p* <0.017 considered significant.

The ETM group reported that their normal life migraine attacks worsened by moderate physical activity in 39/39 (100%) of participants, compared to 55/64 (86%) in the non-ETM group (p = 0.013, ns). Other symptoms (nausea, vomiting, photophobia, phonophobia, unilateral headache) during their normal life attacks did also not differ significantly between the ETM and non-ETM group. Mean age, age of migraine onset, migraine frequency and the presence of high migraine frequency were also equal in the ETM and non-ETM group. Of those reporting to travel by airplane occasionally, in the ETM group 11/39 (28%) reported that airplane travel could provoke an attack, compared to 6/59 (10%) in the non-ETM group (p = 0.02, ns). Frequency of sexual intercourse or Valsalva maneuver as triggers did also not differ in prevalence between ETM and non-ETM group.

## Discussion

To our knowledge this is the first study on the prevalence of ETM and the correlation with detailed characteristics of normal life migraine attacks. The two main findings in this study were; i) ETM attacks were reported by 38% of migraineurs and more than half of those quit the offending sport as a result. ii) During normal life attacks, neck pain as the initial migraine symptom was reported more frequently in migraineurs with ETM attacks compared to migraineurs without ETM attacks.

Several migraine triggers have been reported by migraine patients. Exercise, although not as commonly reported as stress or sleeping disturbance for instance, is one of them [1,6,7]. There is much debate on the existence of these triggers as presumed triggers can co-occur with the migraine just by chance and recall bias is likely to affect retrospective studies. Only few studies reported on patients who were prospectively followed and who were consistently able to trigger attacks by exercising [2,8].

To minimize the possibility that presumed sport provoked attacks in our patients were actually normal life attacks occurring coincidentally during or after exercise, we asked these patients if they had quit the offending exercise because of migraine complaints. This was the case in more than half of patients reporting ETM. Furthermore these patients were able to practice lower intensity exercise in which they experienced no attacks. Both findings make chance as a possible explanation for the relation between exercise and ETM less likely.

If we consider the relation between exercise and migraine to be causal, then what is the underlying pathophysiological mechanism? One possible mechanism is dysfunction of neuropeptides like hypocretin which play a role in regulating sleep and arousal and which are located in brainstem structures which are selectively activated during migraine attacks. Patients often report that sleep is able to end a migraine attack [9], and sleep quality is negatively affected by intense severe exercise [10]. So possibly exercise can influence this hypocretin pathway and thereby trigger attacks.

A second possible mechanism is of cardiovascular origin. Aerobic exercise increases cardiac output and systolic blood pressure. Possibly this rise in cardiac output and systolic blood pressure triggers ETM attacks. This hypothesis is supported by the observation that the use of beta-blockers (which lower the cardiac output and systolic blood pressure) can prevent the occurrence of ETM attacks. It also has been shown that migraineurs have an impaired autonomic control of cerebral vasoreactivity [11], making them more vulnerable for major cardiovascular changes. The fact that in our study the majority of patients with ETM attacks stopped practicing high-intensity exercise, but were able to continue other low-intensity exercise, favors the hypothesis that rises in cardiac output and blood pressure are factors possibly associated with ETM attacks.

A third explanation is based upon an unfavorable energy metabolism. Athletes exercising at an intensity above their aerobic threshold will switch to anaerobic metabolism. The byproduct of this anaerobic exercise is lactate. Magnetic resonance spectroscopy (MRS) showed that higher brain lactate levels were associated with a higher migraine frequency [12]. A different MRS study concluded that energy metabolism in migraineurs is defective, with a slow rate of phosphocreatine recovery after exercise of the muscle in migraineurs [13]. So, while high-intensity exercise leads to a rise in blood lactate, and migraineurs have a defective energy metabolism and a higher brain lactate is associated with a higher migraine frequency, this could explain the triggering of migraine attacks by high-intensity exercise.

We found that neck pain as the initial migraine symptom was more prevalent in migraineurs with ETM. No previous studies reported that specific migraine symptoms were more prevalent in patients experiencing ETM attacks. During the pain phase in migraine the trigeminal complex is activated and neuropeptides are released at the peripheral nerve endings [14]. Neck pain in migraine attacks can be explained by the activation of upper cervical nerve fibers which have their endings in the trigeminal caudalis. Possibly the increased occurrence of neck pain in the ETM group is comparable with allodynia, as patients with allodynia reported more triggers than patients without allodynia [15].

### Limitations

One limitation is the retrospective design of the study, thus recall bias could have influenced results. Furthermore, when patients are questioned if specific events could trigger attacks, their answers are subject to belief and conceptions. However unlike well-known triggers like stress and certain foods, exercise is generally not regarded as a trigger, so this bias might be limited. As this was a clinic based study in a headache center, migraine patients in this study could have been more severely affected than those in the general population. Possibly this may have influenced the lifetime prevalence of ETM attacks reported in this study. In the future prospective diary studies should be conducted, in which every exercise activity (and also other possible confounding triggers) and migraine attack needs to be recorded.

## Conclusion

The lifetime prevalence of ETM attacks among migraineurs was high and was associated with neck pain as initial migraine symptom during normal life migraine attacks. Migraine patients with ETM attacks are generally undertreated and frequently quit high-intensity exercise.

## Abbreviations

ETM: Exercise triggered migraine.

## Competing interests

Both authors report no conflicts of interest.

Authors have no competing interests.

Authors have not received financial support.

## Authors’ contributions

HK designed the study, interviewed all patients, performed the statistical analysis and drafted the manuscript. PLJ participated in the statistical analysis and helped to draft the manuscript. Both authors read and approved the final manuscript.

## Authors’ information

HK works as neurologist at the department of neurology, HagaZiekenhuis Teaching hospital, The Hague, The Netherlands.

PLJV works as sports physician at the department of sports medicine, Medical Center Haaglanden, Leidschendam, The Netherlands.
